# High Expression of *c-kit* mRNA Predicts Unfavorable Outcome in Adult Patients with t(8;21) Acute Myeloid Leukemia

**DOI:** 10.1371/journal.pone.0124241

**Published:** 2015-04-10

**Authors:** Xiaoning Gao, Ji Lin, Li Gao, Ailing Deng, Xiaolin Lu, Yonghui Li, Lili Wang, Li Yu

**Affiliations:** 1 Department of Hematology, Chinese PLA General Hospital, Medical School of Chinese PLA, Beijing, China; 2 Institute of Basic Medicine, Chinese PLA General Hospital, Medical School of Chinese PLA, Beijing, China; 3 Department of Hematology, China-Japan Friendship Hospital, Beijing, China; Queen's University Belfast, UNITED KINGDOM

## Abstract

The reason that a certain subgroup of acute myeloid leukemia (AML) patients with t(8;21) translocation (generating the *AML1/ETO* fusion gene) displays a poor survival remains elusive. The proto-oncogene *c-kit* is expressed in approximately 80% of AML cases. The kinase domain mutation of the *c-kit* gene, one of the most common gain-of-function mutations associated with t(8;21) AML, predicts higher relapse risk and poor prognosis. However, the role of *c-kit* high expression in t(8;21) AML remains poorly understood. Here we evaluated the prognostic significance of *c-kit* expression levels in AML patients. The mRNA expression of *c-kit* was determined by real-time quantitative reverse transcription PCR in 132 adult AML patients. Patients were grouped into quartiles according to *c-kit* expression levels (Q1–Q4, each quartile containing 25% of patients) and divided into *c-kit* high (Q4; n = 33) and *c-kit* low (Q1–Q3; n = 99). High *c-kit* expression was associated with *AML1/ETO-*positive and with *c-kit* mutation. Of note, 35.8% of the *AML1/ETO-*positive AML patients carrying wild-type *c-kit* expressed high levels of *c-kit*, suggesting that other factors are involved in *c-kit* overexpression. High *c-kit* expression was associated with inferior overall and event-free survival in *AML1/ETO-*positive patients and was independently predictive for overall and event-free survival in multivariate analyses in a *c-kit* mutation-independent manner. Thus, high *c-kit* expression serves as a reliable molecular marker for poor prognosis, supporting a pathogenetic role of *c-kit* signaling in *AML1/ETO-*positive AML. *AML1/ETO-*positive patients with high *c-kit* expression might benefit from early treatment modifications and molecular target therapies.

## Introduction

The translocation t(8;21)(q22;q22), generating the *AML1/ETO* fusion gene, is one of the most common structural chromosomal aberrations in patients with acute myeloid leukemia (AML). t(8;21) AML represents a favorable cytogenetic AML subgroup based on its excellent responsiveness to induction chemotherapy and high complete remission (CR) rate [1–3]. However, although the overall disease-free survival reaches about 60% in t(8;21) AML, about 30% to 40% of cases relapse after standard intensive chemotherapy, of which half become treatment resistant [4–7]. Therefore, t(8;21) AML is a heterogeneous disease with poor survival in a subset of patients. Multiple risk factors, including the white blood cell (WBC) count at initial examination, blood platelet count, sex chromosome abnormality and percentage of peripheral blood blasts, have been reported as prognostic factors in t(8;21) AML [5,8–10]. Even so, a certain subgroup of t(8;21) AML patients relapses without showing any above known risk factors. Therefore, stratification of the patients based on more universal risk factors may help identify patients who may benefit from more intensive therapies such as stem cell transplantation during the initial remission period.

Positivity for c-kit expression is present in 80% of AML cases [11], and the frequency of *c-kit* mutations ranges from 13% to 22% in AML with t(8;21) compared with less than 2% in AML cases overall [12,13]. It has been shown that the mutations of *c-kit* gene are a negative prognostic factor correlating higher incidence of relapse and a lower overall survival rate in adult patients as well as in children [13–15]. However, the clinical significance of *c-kit* high expression remains unclear in this subtype of leukemia. Given the pan-expression of c-kit in AML and the prognostic impact of *c-kit* mutations, we analyzed the mRNA expression levels of *c-kit* by quantitative real-time PCR (qPCR) in pretreatment bone marrow samples of 132 adults with AML and evaluated the prognostic significance of *c-kit* expression levels.

In the present study, we show that *c-kit* is highly upregulated in *AML1/ETO*-positive AML. The level of *c-kit* mRNA expression correlates perfectly to *AML1/ETO*. High expression of *c-kit* independently predicts more inferior overall and event-free survival in *AML1/ETO*-positive AML, regardless of *c-kit* mutations.

## Methods

### Ethics Statement

This study was carried out in accordance with principles of Declaration of Helsinki, and was approved by the Human Subject Ethics Committee in Chinese PLA General Hospital. Written informed consent was received from the participants or from the next of kin prior to inclusion in the study.

### Patients and treatments

Bone marrow samples were analyzed from 132 patients with newly diagnosed, untreated AML from Chinese PLA General Hospital. Detection of t(8;21) was routinely accomplished by standard cytogenetic techniques and (or) by FISH using commercially available AML1/ETO probe (Vysis Inc.). Patients were treated with induction therapy consisted of idarubicin (10 mg/m^2^/day × 3)/ daunorubicin (60 mg/m^2^/day × 3)/ mitoxantrone (10 mg/m^2^/day × 3) and cytarabine (100 mg/m^2^/day × 7). Once CR was achieved, consolidation therapy was begun, consisting of intermediate/high dose cytarabine (1.5–2 g/m^2^/12 h on days 1–3) or standard-dose cytarabine-based chemotherapy (idarubicin/ daunorubicin/ mitoxantrone and cytarabine). Allogeneic and autologous hematopoietic stem-cell transplantations were performed in a risk-adapted and priority-based manner.

### RNA isolation and real-time quantitative reverse transcription PCR

Mononuclear cells from bone marrow samples of patients were prepared by Ficoll-Hypaque (Sigma-Aldrich, St Louis, MO) gradient centrifugation. Total RNA was extracted from cells using TRIzol reagent (Invitrogen, Carlsbad, USA). Reverse transcription for obtaining cDNA was performed SuperScript III First-Strand Synthesis System (Invitrogen) according to the manufacturer’s instructions. The expression of *AML1/ETO* and *c-kit* was detected by qPCR using TaqMan Gene Expression Assay (Applied Biosystems, Foster City, CA). Expression of the target genes was determined by absolute quantification method using *ABL1* levels for normalization [16]. The primers and probes specific for *c-kit* and *ABL1* used have been previously described [17]. The following sets of primers and probes were used for *AML1/ETO* detection: Forward: CAAGTCGCCACCTACCACAGA; Reverse: AGCCTAGATTGCGTCTTCACATC; Probe: FAM-CCATCAAAATCACAGTGGAT-NFQ-MGB.

### Statistical analysis

CR was defined as recovery of morphologically normal bone marrow and normal peripheral blood cell count (absolute neutrophil count >1,000/mm^3^ and platelet count >100,000/mm^3^) and no signs or symptoms of the disease or evidence of central nervous system leukemia or other extramedullary infiltration [18]. Relapse was defined by >5% bone marrow blasts, circulating leukemic blasts or development of extramedullary leukemia. Overall survival (OS) was measured from the beginning of therapy until date of death or last follow-up. Event-free survival (EFS) was defined as the time from study entry to first event. An event was defined as failure to achieve a CR, relapse after achieving a CR, or death.

Wilcoxon signed-rank test was selected to determine the difference of *c-kit* expression levels between groups of samples. Spearman’s correlation coefficient (*r*) was used to access the correlation of mRNA levels between *AML1/ETO* and *c-kit*. To compare clinical outcome of patients with different *c-kit* expression levels, the cohort was stratified using the quartile grouping method described previously [19]. Patients were grouped into quartiles according to *c-kit* expression levels (Q1–Q4, each quartile containing 25% of patients) and divided into high *c-kit* (Q4; n = 33) and low *c-kit* (Q1–Q3; n = 99) based on the trend observed in clinical outcome after performing a Cox regression analysis for EFS with *c-kit* quartile grouping as the independent variable. *c-kit* expression ranged between 0.1965 and 10.7182 with the following median expression for each quartile: 0.3604 (Q1), 0.7595 (Q2), 1.0637 (Q3), and 2.9547 (Q4). In this model, *AML1/ETO*-positive patients in the highest *c-kit* quartile showed a significant difference of EFS, as compared to the remaining patients with lower *c-kit* expression levels. The differences in regression coefficients with SE for each quartile were as follows: Q1 versus Q4, -13.692 (SE 405.935), P = 0.973; Q2 versus Q4, -0.615 (SE 0.399), P = 0.123; Q3 versus Q4, = -0.859 (SE 0.386), P = 0.026. Survival curves were generated using the Kaplan-Meier method and the log-rank test was used to compare survival between groups. Clinical features across groups were compared using the 2-sided Fisher exact test for categorical data and the nonparametric Mann-Whitney U test for continuous variables. The Cox proportional hazards model with stepwise forward selection were constructed to determine whether *c-kit* expression was associated with outcome when adjusting for other prognostic variables. The full multivariate model used the variables significant at a 10% level in univariate analysis, including *c-kit* expression (low *vs*. high), *c-kit* mutation status (mutation *vs*. wild-type), white blood count (10 × 10^9^/L increase), bone marrow blasts (10% increase), age (10-year increase), cytarabine-based chemotherapy (high- *vs*. standard-dose), hematopoietic stem-cell transplantations (Allogeneic- *vs*. no, autologous- *vs*. no) and CR achievement (1 *vs*. ≥ 2 courses). The possible influence of sample bias on the results and the stability of the model were examined by bootstrap resampling method [20]. A total of 1000 bootstrap samples were generated for each analysis. Cox regression was run separately on these 1000 samples to obtain robust estimates of the standard errors of coefficients, and hence the *P* values and 95% confidence intervals of the model coefficients. SPSS 20.0 software was used to process the data. A P value of less than 0.05 was chosen as a threshold for statistical significance.

## Results

### Correlations of *c-kit* expression with t(8;21) AML

**Fig 1:**
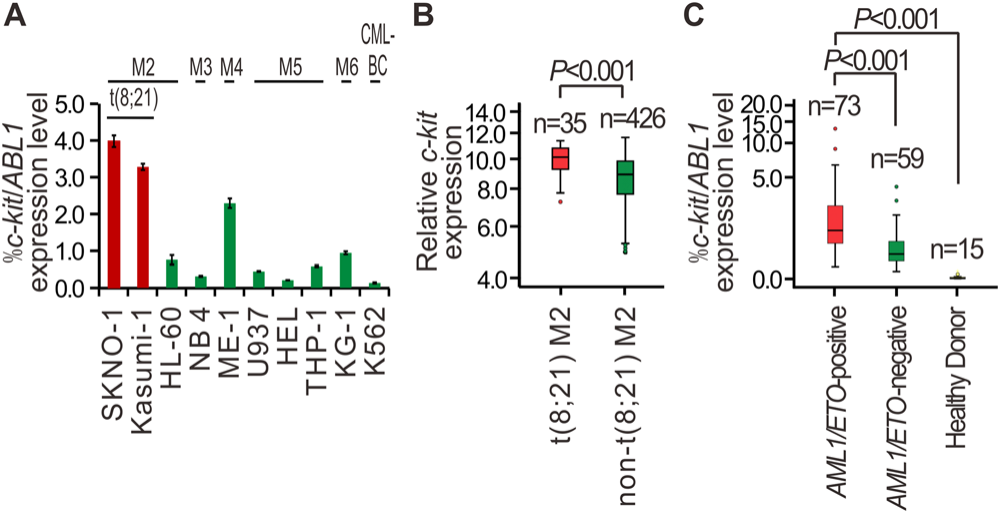
Selective high expression of *c-kit* in *AML1/ETO*-positive AML cell lines and patients. **(A)** qPCR showing *c-kit* expression in myeloid leukemia cell lines. CML-BC: chronic myeloid leukemia in blast crisis. Bars indicate the mean±SEM from three independent experiments. *ABL1* levels were measured for normalization. **(B)** Normalized *c-kit* expression in pretreatment samples of 461 patients with *de novo* AML (GEO database, GSE6891). The gene expression was determined using gene-expression arrays (Affymetrix HGU133 Plus 2.0 GeneChips), which reflected by the intensity of hybridization of labeled mRNA to the gene chip. Median values are depicted by the horizontal lines. The Mann-Whitney U test was used to compare expression levels between groups. **(C)** qPCR showing *c-kit* expression level in bone marrow samples from untreated AML patients at diagnosis and healthy donors. *ABL1* levels were measured for normalization. Median values are depicted by the horizontal lines. The Mann-Whitney U test was used to compare expression levels between groups.

We initially detected *c-kit* mRNA expression levels in 10 myeloid leukemia cell lines using qPCR (Fig 1A) and then analyzed *c-kit* expression pattern in a previously published microarray dataset GSE6891 [21], which included gene expression profiles of 461 blood or bone marrow samples from AML patients (Fig 1B). The result showed *c-kit* was significantly upregulated in t(8;21) AML cell lines and patients, as compared to t(8;21)-negative subtypes. To further confirm the selectively high expression of *c-kit* in t(8;21) AML cells, using qPCR analysis, we measured *c-kit* levels in bone marrow mononuclear cells from 132 newly diagnosed AML patients and 15 healthy donors (S1 Dataset). As shown in Fig 1C, *c-kit* mRNA levels were significantly elevated in bone marrow samples from *AML1/ETO*-positive patients (*n =* 73), as compared to those from *AML1/ETO-*negative (*n =* 59) and healthy controls (*n =* 15; both *P =* 0.000).

### *c-kit* expression with respect to clinical and biologic characteristics

**Fig 2:**
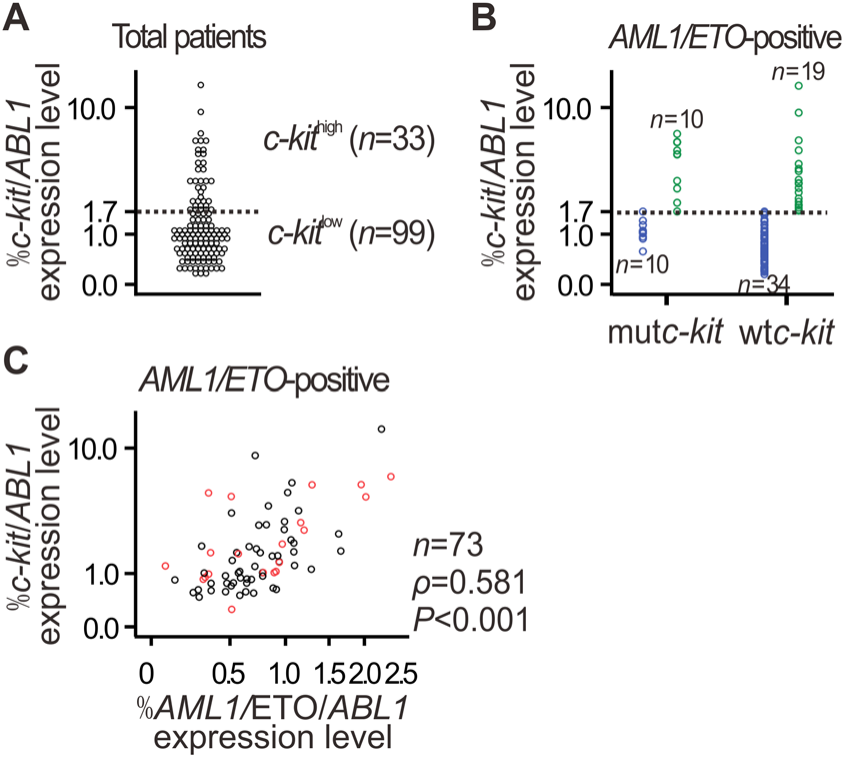
Positive correlation between *AML1/ETO* and *c-kit* expression levels in *AML1/ETO*-positive AML. **(A)** The patients described in Fig 1C were divided into high and low *c-kit* expression groups, which described in detail in statistical analysis. The threshold is depicted as a dashed line. Patient characteristics are described in Table 1. **(B)** Stratification of *AML1/ETO*-positive AML patients with high and low *c-kit* expression according to *c-kit* mutation status. **(C)** Correlation between *c-kit* and *AML1/ETO* levels in *AML1/ETO*-positive AML patients was assessed by the Spearman rank correlation coefficient. Gene expression was detected by qPCR. *ABL1* levels were measured for normalization. Black circles indicate AML patients carrying wt*c-kit*.

For further determination of the correlation between *c-kit* and *AML1/ETO* expressions, patients were divided into two groups, high and low, according to *c-kit* mRNA levels (Fig 2A). Correlation analyses between *c-kit* levels and the clinical and biologic features of the patients revealed that high *c-kit* expression was significantly associated with *AML1/ETO-*positive (*P =* 0.000) or *c-kit* mutation (mut*c-kit*) (*P =* 0.019) in the overall cohort (Table 1). Of note, high expression of *c-kit* is independent of mut*c-kit* (*P>*0.05; Table 1) in the *AML1/ETO*-positive cohort. Actually, in *AML1/ETO*-positive patients carrying wild-type *c-kit* (wt*c-kit*), 19 of 53 (35.8%) presented high level of *c-kit* expression (Fig 2B). Further, there was a strong positive correlation between *AML1/ETO* and *c-kit* expression levels in the *AML1/ETO*-positive patients, especially in those carrying wt*c-kit* (n = 53, *ρ* = 0.601, *P*<0.001) (Fig 2C).

**Table 1:** Comparison of clinical and biologic variables of AML patients according to *c-kit* expression.

Characteristic	Overall cohort (*n =* 132)		*P* value	*AML1/ETO-*positive (*n =* 73)		*P* value	*AML1/ETO-positive and wtc-kit* (*n =* 53)		*P* value
	*c-kit* ^high^	*c-kit* ^low^		*c-kit* ^high^	*c-kit* ^low^		*c-kit* ^high^	*c-kit* ^low^	
Patients no.	33	99		29	44		19	34	
Age ^a^	33 (11–60)	36 (12–84)	0.245	33.0 (11–60)	26 (12–82)	0.875	34 (11–59)	26 (12–62)	0.676
*Sex*, *no*. *(%)*			0.534			0.655			0.910
Male	22 (66.7)	60 (60.6)		18 (62.1)	25 (56.8)		12 (63.2)	22 (64.7)	
Female	11 (33.3)	39 (39.4)		11 (37.9)	19 (43.2)		7 (36.8)	12 (35.3)	
WBC (× 10^9^/L) ^a^	12.7 (2.7–290.0)	13.1 (0.3–362.0)	0.870	12.2 (2.7–55.9)	14.5 (1.5–76.3)	0.423	11.0 (3.5–37.4)	14.6 (2.1–76.3)	0.349
Bone marrow blasts (%) ^a^	64.0 (21.2–92.0)	55.0 (7.4–97.0)	0.084	65.0 (36.8–92.0)	53.1 (7.4–95.0)	0.038	64.0 (36.8–92.0)	52.1 (7.4–95.0)	0.040
*FAB subtypes*, *no*. *(%)*			0.546						
M2	30 (26.1)	85 (73.9)		29 (39.7)	44 (60.3)		19 (35.8)	34 (64.2)	
M3	0 (0)	2 (100)							
M4	3 (33.3)	6 (66.7)							
M5	0 (0)	4 (100)							
M6	0 (0)	2 (100)							
*AML1/ETO status* ^*b*^, *no*. *(%)*			0.000						
*AML1/ETO* positive	29 (87.9)	44 (44.4)							
*AML1/ETO* negative	4 (12.1)	55 (55.6)							
*c-kit mutation status* ^*c*^, *no*. *(%)*			0.019			0.270			
mut*c-kit*	10 (34.5)	10 (13.9)		10 (34.5)	10 (22.7)				
wt*c-kit*	19 (65.5)	62 (86.1)		19 (65.5)	34 (77.3)				
*Chemotherapy* ^*c*^, *no*. *(%)*			0.575			0.752			0.376
High-dose cytarabine-based	9 (37.5)	19 (31.1)		9 (45.0)	10 (50.0)		6 (37.5)	8 (53.5)	
Standard-dose cytarabine-based	15 (62.5)	42 (68.9)		11 (55.0)	10 (50.0)		10 (62.5)	7 (46.7)	
*HSCT*, *no*. *(%)*			0.895			0.899			0.972
Allo-HSCT	8 (24.2)	27 (27.3)		6 (20.7)	8 (18.2)		3 (15.8)	6 (17.6)	
Auto-HSCT	3 (9.1)	7 (7.1)		3 (10.3)	6 (13.6)		2 (10.5)	4 (11.8)	
No HSCT	22 (66.7)	65 (65.7)		20 (69.0)	30 (68.2)		14 (73.7)	24 (70.6)	
*CR* ^*c*^, *no*. *(%)*			0.297			0.606			0.611
1 course	15 (62.5)	31 (50.0)		13 (65.0)	12 (57.1)		11 (68.8)	9 (60.0)	
≥ 2 courses	9 (37.5)	31 (50.0)		7 (35.0)	9 (42.9)		5 (31.3)	6 (40.0)	

^a^ Values represent median (range).

^b^ The AML/ETO status was confirmed using qPCR analysis.

^c^ Information is not available in some cases. HSCT, hematopoietic stem cell transplantation.

### *c-kit* expression and outcome in overall cohort of AML patients

**Fig 3:**
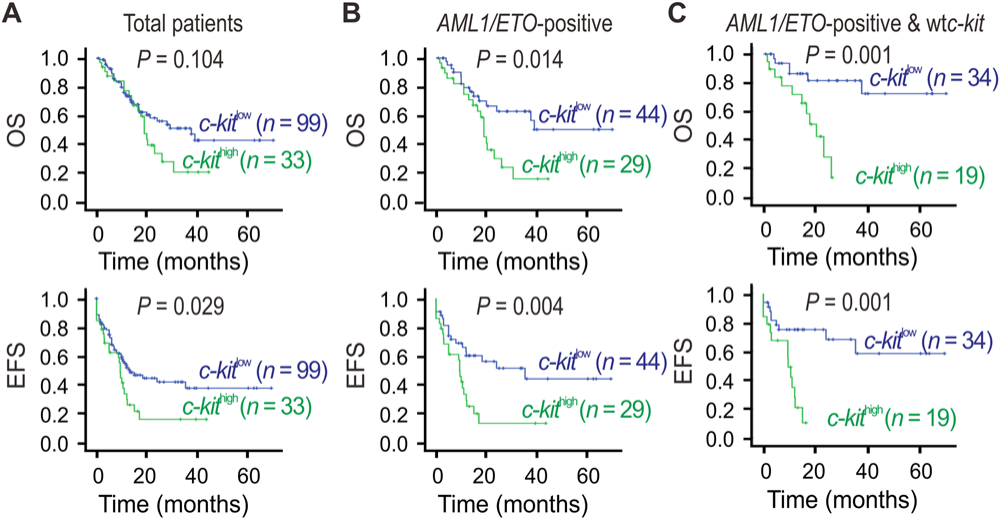
Higher expression of *c-kit* in *AML1/ETO-*positive AML predicts more inferior prognosis. **(A-C)** Kaplan-Meier estimate for OS and EFS in indicated patient subgroups. Survival curves were compared using log-rank test.

Prognostic significance of *c-kit* expression analysis was first performed in the whole population (Fig 3A). High *c-kit* expression was not a poor prognostic factor for OS. In contrast, EFS were significantly shorter in the patients with high *c-kit* expression (99 patients) compared with the patients with low *c-kit* expression (33 patients). The estimated 3-year OS rates of *c-kit* high patients were 16.6% plus or minus 7.7% (median = 9.4 months) versus 38.2% plus or minus 6.8% (median = 13.4 months) for *c-kit* low patients (*P =* 0.029; Fig 3A).

### *c-kit* expression and survival in *AML1/ETO*-positive AML patients

A total of 78 patients with *AML1/ETO* expression were separately analyzed. Patients expressing high *c-kit* levels had a shorter OS (16.1%±9.2% at 3 years, median = 19.0 months *vs*. 63.0%±8.4% at 3 years, median *=* not reached, *P =* 0.014), and EFS (13.5%±7.8% at 3 years, median *=* 9.4 months *vs*. 51.7%±8.8% at 3 years, median *=* 35.3 months, *P =* 0.004) than patients expressing low *c-kit* levels (Fig 3B). Notably, further stratification of patient groups based on *c-kit* mutation status improved the predictive capability of *c-kit* for both OS and EFS (Fig 3C). In the *AML1/ETO*-positive AML patients carrying wt*c-kit* [*n =* 53, about 73% (53/73) of the entire *AML1/ETO*-positive patients], those expressing high *c-kit* levels had a shorter OS (14.2%±12.4% at 3 years, median *=* 20.3 months *vs*. 81.6%±7.6% at 3 years, median *=* not reached, *P =* 0.001) and EFS (10.5%±9.1% at 3 years, median *=* 9.5 months *vs*. 58.6%±12.2% at 3 years, median *=* not reached, *P =* 0.001) than those expressing low *c-kit* levels.

### Multivariate analysis and *c-kit* expression

According to the prognostic value of *c-kit* expression in AML patients, *c-kit* expression was entered into a multivariate model in addition to factors significantly associated with prognosis in univariate analysis (Table 2). In the overall cohort, *c-kit* expression was not independently predictive for OS or EFS. In contrast, in the *AML1/ETO*-positive patients, *c-kit* expression was an independent prognostic parameter for both OS (*P =* 0.049) and EFS (*P =* 0.033). Remarkably, in the *AML1/ETO*-positive and wt*c-kit* subgroup, high *c-kit* expression was the only parameter with prognostic significance for OS (*P =* 0.003) and EFS (*P =* 0.002). To further investigate the stability of the classical multivariate Cox models shown in Table 2, we conducted a bootstrap resampling procedure to calculate the standard errors of regression coefficients along with corresponding *P* values and 95% confidence intervals of the model. We found no significant differences in *P* values of regression coefficients between the classical Cox regression and bootstrap Cox regression models (Table 2).

**Table 2:** Multivariate analysis for OS and EFS and bootstrap resampling for variable in the multivariate model.

Patients	Variable ^a^	Cox regression		Bootstrap resampling			
		*P* value	Hazard Ratio (95.0% CI)	Regression Coefficient	Std. Error	Bootstrap^b^	
						*P* value	95.0% CI
*overall cohort*							
	*OS*						
	I/HD *vs*. SD cytarabine-based chemotherapy	**0.002**	0.261 (0.110–0.618)	-1.311	0.439	**0.001**	-2.294 – -0.562
	*EFS*						
	I/HD *vs*. SD cytarabine-based chemotherapy	**0.002**	0.321 (0.155–0.666)	-1.068	0.361	**0.002**	-1.857 – -0.467
*AML1/ETO-positive patients*							
	*OS*						
	*c-kit* high *vs*. *c-kit* low	**0.049**	2.810 (1.003–7.872)	1.033	0.995	**0.048**	-0.039–2.483
	I/HD *vs*. SD cytarabine-based chemotherapy	**0.012**	0.233 (0.075–0.721)	-1.458	1.214	**0.008**	-3.157 – -0.411
	*EFS*						
	*c-kit* high *vs*. *c-kit* low	**0.033**	2.739 (1.086–6.910)	1.008	0.809	**0.030**	0.054–2.346
	I/HD *vs*. SD cytarabine-based chemotherapy	**0.013**	0.298 (0.115–0.771)	-1.211	0.609	**0.005**	-2.483 – -0.400
*AML1/ETO-positive and wtc-kit patients*							
	*OS*						
	*c-kit* high *vs*. *c-kit* low	**0.003**	5.086 (1.732–14.933)	1.626	0.651	**0.005**	0.637–3.222
	*EFS*						
	*c-kit* high *vs*. *c-kit* low	**0.002**	4.093 (1.695–9.888)	1.409	0.490	**0.004**	0.599–2.527

^a^ Variables considered for model inclusion were: *c-kit* expression (high *vs*. low), *c-kit* mutation status (mutation *vs*. wild-type), WBC count (10×10^9^/L increase), bone marrow blasts (10% increase), age (10-year increase), cytarabine-based chemotherapy (intermediate/high dose- *vs*. standard-dose), HSCT (allo- *vs*. no, auto- *vs*. no) and CR achievement (1 *vs*. ≥ 2 courses). Only variables significantly associated with outcomes in univariate analysis were included in the multivariate model.

^b^ bootstrap results are based on 1000 bootstrap samples. SD, standard dose; I/HD, intermediate/high dose.

## Discussion

Although *AML1/ETO*-positive AML patients achieve an initial complete remission, most of them relapse for undefined reasons. The present investigation was undertaken to address why a certain subgroup of *AML1/ETO-*positive AML patients displays a poor survival. Our findings establish *c-kit* as a reliable molecular marker, which identify patients with an inferior prognosis in an otherwise prognostically favorable *AML1/ETO-*positive AML.

In analyzing *c-kit* expression in AML patients, we found that *c-kit* was highly elevated in *AML1/ETO-*positive patients as compared to *AML1/ETO-*negative ones. When *AML1/ETO-*positive patients were divided into two groups regarding *c-kit* expression levels, we evidenced the positive correlation between *AML1/ETO* and *c-kit*. As a leukemia-initiating transcriptional factor, AML1/ETO is not sufficient in itself to induce leukemogenesis [22]. The *c-kit* proto-oncogene plays a very important role in the occurrence and development of multiple tumor types through regulating cellular differentiation and proliferation [23–26]. Mutations of the activation loop of c-kit kinase domain, leading to constitutive activation of the c-kit receptor kinase and thereby its downstream signaling transduction pathways, co-operate with AML1/ETO to cause leukemia as a critical genetic event [27]. Mutations of *c-kit* were associated with a higher relapse risk and a lower overall survival rate in t(8;21) AML patients [13–15,28]. Especially, AML1/ETO9a, a spliced isoform of AML1/ETO [29], correlates with *c-kit* overexpression/mutations and indicates poor disease outcome in the FAB-M2 subtype of t(8;21) AML [30]. However, the correlation between *AML1/ETO* and *c-kit* high expression and their biological importance and functional properties in t(8;21) AML remains poorly understood.

In the present study, the fact that high *c-kit* expression was also detected in a considerable part of *AML1/ETO-*positive AML patients carrying wt*c-kit*, indicating that other factors, except for mut*c-kit*, might contribute to the elevated expression of *c-kit*. Actually, the statistically significant positive correlation between *AML1/ETO* and *c-kit* mRNA levels in AML patients, especially in those carrying wt*c-kit*, suggests a potential regulatory interaction between *AML1/ETO* and *c-kit*. According to the data of our group, AML1/ETO can inhibit the expression of tumor suppressor gene *microRNA-193a* by inducing DNA hypermethylation in the promoter region of *microRNA-193a* [31]. The *microRNA-193a* can inhibit the overexpression of *c-kit* oncogene in *AML1/ETO*-positive leukemia cells [32]. Therefore, it is very likely that *c-kit* is a target of AML1/ETO. Actually, a previous study has proved that induced expression of *AML1/ETO* in leukemia cells significantly up-regulates both mRNA and protein levels of *c-kit* gene [33]. Together, these findings support the notion that c-kit could be a downstream target of AML1/ETO. Further mechanistical analysis should be done to unveil the molecular regulation pathway between AML1/ETO and c-kit.

While t(8;21) AML have a relatively good prognosis, about 30% to 40% of relapse rate has been observed for unknown reasons [4–7]. In this study, we have identified high *c-kit* expression as an independent prognostic factor associated with an inferior EFS and OS in *AML1/ETO-*positive AML patients. It is worth noting that *c-kit*-associated inferior prognosis in *AML1/ETO*-positive AML patients is *c-kit* mutation-independent because the predictive capability of *c-kit* for both OS and EFS was further improved in the subgroup of patients carrying *AML1/ETO* and wt*c-kit*. Certain reports have suggested that the number of *AML1/ETO* transcripts could serve as an indicator for relapse, since higher *AML1/ETO* transcript at diagnosis and a limited reduction of *AML1/ETO* transcript at the time of achieving complete remission predict a higher risk of relapse [34–35]. However, the underlying mechanisms are still not known. In the present study, the significant correlation between *AML1/ETO* and *c-kit* expressions or between the high *c-kit* expression and the poor outcome in *AML1/ETO*-positive patients provides an alternative explanation for why high *AML1/ETO* transcript predicts high relapse risk and supports the critical contribution of *c-kit* to *AML1/ETO*-driven leukemia.

In conclusion, we identified high *c-kit* expression as an independent adverse prognostic factor in adult *AML1/ETO*-positive AML, thereby serving as a useful marker for poor prognosis. Our results demonstrate the role of c-kit, which is essential to identify therapeutic targets that are specific to *AML1/ETO-*positive AML.

## Supporting Information

S1 Dataset Individual information of patients and healthy donors. Individual clinical characteristics and *c-kit* mRNA expression levels of 132 newly diagnosed AML patients (A) and 15 healthy donors (B).
(XLSX)
